# Supplementary material for: Gold nanoparticle-enhanced radiotherapy: Dependence of the macroscopic dose enhancement on the microscopic localization of the nanoparticles within the tumor vasculature
Source: PLoS One. 2024 Jul 5;19(7):e0304670. doi: 10.1371/journal.pone.0304670 (PMC11226116; doi:10.1371/journal.pone.0304670)
Supplement: Attachment — Submitted filename: LIST OF MAJOR CHANGES.docx [file pone.0304670.s001.docx]

**LIST OF MAJOR CHANGES**

All changes are highlighted in yellow in the revised manuscript.

1. The following paragraph has been added in section 2.a “Tumor vasculature model” in the revised manuscript: *“While the tumor vasculature model is small in size, a necessity dictated by its high resolution, the fact that it correctly reproduces the ratio of vasculature-to-tissue volume, that it is composed of biological materials, that it is embedded in a soft tissue sphere in order to achieve electronic equilibrium inside of it, and that it is, nevertheless, large enough to allow us to calculate absorbed dose at the dimensions typically used in radiotherapy treatment planning, makes this model suitable for the purpose of determining the effect that the presence of GNPs have on the absorbed dose imparted not only to the tumor tissue but also to the blood vessel walls.”*
2. The following paragraph has been added in section 2.b in the revised manuscript: *“While it is likely that once GNPs extravasate from the vasculature and diffuse in the surrounding tissue a concentration gradient would form, this would only be the case if the blood vessels are rectilinear and fairly parallel to each other. For the inherently random tumor vasculature, we decided that the most representative approach would be to consider a uniform spatial distribution of the GNPs in the tumor tissue.”*
